# Reorientation technique has benefits in bone marrow aspiration of stem cells

**DOI:** 10.1038/s41598-022-15019-7

**Published:** 2022-07-08

**Authors:** Christof Pabinger, Dietmar Dammerer, Harald Lothaller, Georg Stefan Kobinia

**Affiliations:** 1 IRM-Institute for Regenerative Medicine, Plüddemanngasse 45, 8010 Graz, Austria; 2 Medical University of Innsbruck, Christof Probst Platz 1, 6020 Innsbruck, Austria; 3 Department of Orthopaedics and Traumatology, University Hospital Krems, Krems, Austria; 4 Statistics, University of Music and Performing Arts, Leonhardstraße 15, 8010 Graz, Austria

**Keywords:** Haematopoietic stem cells, Mesenchymal stem cells, Stem-cell research

## Abstract

We treated patients with osteoarthritis of the knee using injections of bone marrow aspirate concentrate (stem cell therapy). Since multiple controversial harvesting methods using different sites, needles, volumes and techniques have been described, we aimed to compare those methods. Four different harvesting sites at the iliac crest, three different types of needles, three different types of volumes and two different harvesting techniques were compared in 48 bone marrow aspirations. The conventional technique (Group 1) was compared with a reorientation technique (Group 2). The number of leucocytes and CD34 + cells and the viability in bone marrow aspirate (BMA) were analysed with a CytoFLEX Flow Cytometer. The reorientation technique showed significantly higher cell counts than the conventional technique in all parameters. Leucocytes per nl increased from 5 ± 2 to 12 ± 4 (p < .001), and CD 34 + cells per μl increased from 40 ± 40 to 140 ± 98 (p = .003). There was no difference between anterior and posterior harvesting at the iliac crest or between use of a thick and use of a thin needle. Use of the reorientation technique, compared to employing the conventional technique, has a significant advantage since the number of leucocytes and CD34 + cells can be tripled. For the use of bone marrow aspirate in the case of arthritis, it might therefore be a future option to harvest a maximum cell yield through the new reorientation technique and to omit centrifugation. However, the clinical relevance of these findings remains the subject of future studies.

Level of Evidence: Level I.

Clinical relevance: Enhanced technique of BMA for knee surgeons to ensure the maximum cell yield for stem cell therapy in regenerative medicine.

## Introduction

Point-of-care harvest and application of residence stem cells are practical and cost-effective^1^.

Bone marrow aspiration is an effective means of harvesting various tissue progenitor cells but is associated with dilution with peripheral blood. Patterson found that 63.5% of all connective tissue progenitor cells within iliac cancellous bone resided on the trabecular surface^2^. It is therefore a difficult task to obtain the maximum number of cells, and no consensus exists regarding which method is superior to the others. Although bone marrow aspiration has been described for more than 70 years, a review of 1580 studies revealed that they reported information on only 42% of the variables included within established minimum reporting standards^3,4^. Therefore, it was no earlier than 2020 when the age-dependent normal values of leukocytes and CD34 + cells in human bone marrow were published for the first time on a larger series of patients^5^. For the first decades, bone marrow aspirations were mainly used for diagnostic reasons. With the rise of regenerative medicine and level II evidence for osteoarthritis, stem cell therapy is gaining increasing importance^6–8^. This necessitates an optimal harvesting procedure, where a maximum yield of cells can be retrieved with minimally invasive procedures. However, the optimal cell composition of the aspirate for osteoarthritis treatment is still unknown. A consensus exists among surgeons that a high cell number and a high viability should be achieved, and therefore, different harvesting techniques have been described^9–13^. In general, the technique has deteriorated over the last decades^14^. Although different therapeutic protocols for stem cell preparation in the clinical orthopaedic literature exist^15^, it is not fully understood which type of (mononuclear) cells (CD34 + , mesenchymal stem cells [MSCs], etc.) can be linked to clinical success. Furthermore, MSCs can only be measured by indirect means with several other markers and using cultivation and evaluation with colony forming units, whereas CD34 + cells can be measured directly. Both cell types represent a small proportion of bone marrow-derived mononuclear cells. To evaluate a new harvesting technique, it is therefore recommended to exclude as many pre- and postanalytic confounders from the laboratory as possible. Therefore, the measurement of CD34 + cells alone, which contribute to approximately 1% of all bone marrow-derived mononuclear cells^16^, instead of the indirect measurement and cultivation of MSCs is sufficient to identify the number of mononuclear cells.

In recent years, a variety of different medical devices for bone marrow harvesting have emerged, and to date, the following four questions (site, needle, volume, technique) remain open, and the respective papers remain controversial:

Q1: What is the best SITE for bone marrow aspiration? Pierini stated that the posterior iliac crest outperformed the anterior iliac crest, but this referred only to the yield of colony-founding connective-tissue progenitors; all other variables did not differ significantly^17^. McLain found no difference in the nucleated cell count between the iliac crest and the vertebra and that the biologic activity and prevalence of the connective-tissue progenitor cells of vertebrae were comparable with those of cells from the iliac crest^18^. Carceles showed that the concentration of mononucleated cells and the potential for differentiation of cells from the iliac crest were higher than those from cells harvested in the distal femur and the proximal tibia^19^.

However, in clinical practice, it makes a difference if the cells can be harvested from the ventral part of the iliac crest in the supine position or if the patient has to be in the prone or lateral position, where airway control is more difficult, to reach the spina iliaca posterior. Hernigou indicated safe and less safe sectors of the iliac bone^20^.

Q2: Which NEEDLE is the best? Diameters from most bone marrow aspiration needles range from 1 to 3 mm, and in some cases, a blunt mandrin is used. Some needles are 1.5 cm long, while others are up to 6 cm long. Most needles have an aspiration hole at the tip of the needle, whereas the Marrow Cellution® needle has a blunt tip and aspirates through multiple lateral holes in the side. However, this specific needle has to be inserted deep inside the bone using a hammer, has a thick diameter and is very costly^9^. In contrast, an Argon® needle costs approximately 100 times less and can be easily placed into the bone by hand since it is thinner. Glinn found a trend towards fewer aspiration artefacts in manual bone marrow biopsy specimens compared to specimens obtained with a motorized device^12^.

Q3: Which VOLUME should be retrieved? 1997 Muschler favoured a small-volume technique^21^. However, this was contradicted by Fennema in 2009, who published that at least 8 ml should be aspirated to reduce the risk of low cell numbers and that “from the same site, a second aspiration or an aspirate of > 10 mL can be drawn without any loss of biological quality due to dilution with peripheral blood”^22^. In contrast, in 2013, Hernigou again highlighted the benefits of a small volume and small syringe for bone marrow aspirations of mesenchymal stem cells^23^. Abazovic reported that he generally uses 80 ml aspirated with a 3 mm thick needle, since he wants to collect as many cells as possible^24^.

Q4: Which TECHNIQUE should be used? Only a few publications describe the bone marrow aspiration technique in detail; moreover, in nearly 60% of all publications, this information is either absent or insufficient^4^. Chahla described the aspiration of 2 × 30 ml in a single-site technique^25^. Abazovic reported 2 × 50 ml collection from the same site^24^. Olivier did not find a significantly higher cell yield with the multiple-site technique than with the single-site technique, but only 6 patients were included^13^. Inconsistent results regarding the clinical outcome in stem cell publications in general and differences in cell count between the different studies may be explained by the heterogeneity of bone marrow harvesting techniques^26^. Recent publications demand precise guidelines for the techniques used^4,11,26,27^. However, little is known about how to do this correctly.

Therefore, the objective of the present study was to find the technique with the maximum yield of CD34 + cells and the maximum viability of cells. We therefore compared these 4 parameters in a prospective blinded study involving the following: (1) Different sites of aspiration at the iliac crest (anterior versus posterior). (2) Miscellaneous types of needles (diameter 1–3 mm, lateral versus tip suction, 1€ to 600€). (3) Various volumes of syringes (10–30 ml). (4) Two different aspiration techniques (conventional technique versus reorientation technique).

## Results

The new reorientation technique (Group 2) showed significantly higher cell counts (p = 0.001 to 0.003) than the conventional technique (Group 1) in all parameters, regardless of the needle (Argon and Arthex) and volume (Arthrex and Luerloc) employed; see Tables 2, 3 and 4.

There was no significant difference in patient epidemiology between the two patient groups (see Table 1).

**Table 1:** Epidemiology of the 2 groups showed good comparability.

	Group 1	Group 2	p
Technique	“Conventional”	“Reorientation”	
Bone marrow harvest site	Ventral and dorsal	Ventral	
Puncture technique	No reorientation	Needle reorientation	
Volume	Large volume	Multiple small volume	
**Demography**			
Age (years)	50 ± 16 (22–86)	52 ± 18 (25–76)	n.s
Sex (m/w)	6/3	5/3	n.s
Weight (kg)	69 ± 16 (44–92)	64 ± 15 (46–85)	n.s
Height (m/w)	171 ± 9 (151–185)	169 ± 15 (150–186)	n.s
Diagnosis: Arthritis knee	100%	100%	n.s
Follow up rate	100%	100%	n.s
Number of patients	9	8	n.s
Bone marrow punctures	32	16	

### Q1: site of aspiration

No significant difference regarding the BMA cell count could be found between anterior or posterior harvesting of the iliac crest: The cell count, viability and percentage of CD + cells of anterior and posterior harvesting correlated strongly within the same patient (who served as his own control), see Table 2. Since this was proven in the standard technique (Group 1), the patients receiving the reorientation technique (Group 2) were only punctured from the anterior aspect (and not from both the anterior and the posterior aspects) to minimize harm.

**Table 2:** Group 1 (conventional technique, single orientation): Comparison of different needles, different syringes and different anatomic locations (anterior versus posterior and left versus right).

Group	Patient #	Orientation	Volume	Centrifuge	Needle	Syringe/volume	Location	Side	Leu/nl	Viability CD34 (%)	CD34 /µl
1	1	Single	Big	Arthrex angel	Argon	Luerloc (6 $\times$ 10 ml)	Anterior	Right	7	71	126
1	2	Single	Big	Arthrex angel	Argon	Luerloc (6 $\times$ 10 ml)	Anterior	Right	6	63	26
1	3	Single	Big	Arthrex angel	Argon	Luerloc (6 $\times$ 10 ml)	Anterior	Right	10	72	158
1	4	Single	Big	Arthrex angel	Argon	Luerloc (6 $\times$ 10 ml)	Anterior	Right	6	63	14
1	5	Single	Big	Arthrex angel	Argon	Luerloc (6 $\times$ 10 ml)	Anterior	Right	3	68	13
1	6	Single	Big	Arthrex angel	Argon	Luerloc (6 $\times$ 10 ml)	Anterior	Right	2	85	13
1	7	Single	Big	Arthrex angel	Argon	Luerloc (6 $\times$ 10 ml)	Anterior	Right	2	83	29
1	8	Single	Big	Arthrex angel	Argon	Luerloc (6 $\times$ 10 ml)	Anterior	Right	3	80	17
1	9	Single	Big	Arthrex angel	Argon	Luerloc (6 $\times$ 10 ml)	Anterior	Right	3	78	6
								Mean	5 ± 3	74 ± 8	45 ± 53
1	1	Single	Big	Arthrex angel	Argon	Luerloc (6 $\times$ 10 ml)	Posterior	Right	11	59	103
1	2	Single	Big	Arthrex angel	Argon	Luerloc (6 $\times$ 10 ml)	Posterior	Right	4	59	32
1	3	Single	Big	Arthrex angel	Argon	Luerloc (6 $\times$ 10 ml)	Posterior	Right	8	77	106
1	4	Single	Big	Arthrex angel	Argon	Luerloc (6 $\times$ 10 ml)	Posterior	Right	6	75	28
1	5	Single	Big	Arthrex angel	Argon	Luerloc (6 $\times$ 10 ml)	Posterior	Right	7	84	18
1	6	Single	Big	Arthrex angel	Argon	Luerloc (6 $\times$ 10 ml)	Posterior	Right	2	82	20
1	7	Single	Big	Arthrex angel	Argon	Luerloc (6 $\times$ 10 ml)	Posterior	Right	6	86	5
								Mean	6 ± 3	75 ± 11	45 ± 39
1	1	Single	Big	Arthrex angel	Arthrex	Arthrex (2 $\times$ 30 ml)	Anterior	Left	7	72	70
1	2	Single	Big	Arthrex angel	Arthrex	Arthrex (2 $\times$ 30 ml)	Anterior	Left	6	58	55
1	3	Single	Big	Arthrex angel	Arthrex	Arthrex (2 $\times$ 30 ml)	Anterior	Left	10	77	98
1	4	Single	Big	Arthrex angel	Arthrex	Arthrex (2 $\times$ 30 ml)	Anterior	Left	6	75	25
1	5	Single	Big	Arthrex angel	Arthrex	Arthrex (2 $\times$ 30 ml)	Anterior	Left	3	73	13
1	6	Single	Big	Arthrex angel	Arthrex	Arthrex (2 $\times$ 30 ml)	Anterior	Left	2	82	43
1	7	Single	Big	Arthrex angel	Arthrex	Arthrex (2 $\times$ 30 ml)	Anterior	Left	2	87	3
1	8	Single	Big	Arthrex angel	Arthrex	Arthrex (2 × 30 ml)	Anterior	Left	3	78	11
1	9	Single	Big	Arthrex angel	Arthrex	Arthrex (2 × 30 ml)	Anterior	Left	3	79	6
								Mean	5 ± 3	76 ± 8	36 ± 31
1	1	Single	Big	Arthrex angel	Arthrex	Arthrex (2 × 30 ml)	Posterior	Left	8	70	73
1	2	Single	Big	Arthrex angel	Arthrex	Arthrex (2 × 30 ml)	Posterior	Left	7	69	54
1	3	Single	Big	Arthrex angel	Arthrex	Arthrex (2 × 30 ml)	Posterior	Left	20*	73	276*
1	4	Single	Big	Arthrex angel	Arthrex	Arthrex (2 × 30 ml)	Posterior	Left	5	74	35
1	5	Single	Big	Arthrex angel	Arthrex	Arthrex (2 × 30 ml)	Posterior	Left	7	88	13
1	6	Single	Big	Arthrex angel	Arthrex	Arthrex (2 × 30 ml)	Posterior	Left	2	79	15
1	7	Single	Big	Arthrex angel	Arthrex	Arthrex (2 × 30 ml)	Posterior	Left	4	88	20
								Mean	5 ± 2	77 ± 7	35 ± 22
Group 1: no significant differences between4 groups or pooled data (anterior/posterior, left/right)								Mean all patients:	5 ± 2	75 ± 9	40 ± 40 * values omitted (outliers)

There was no difference between the respective groups. The absolute number of cells was lower than that in Group 2 (multiple orientation technique).

### Q2: needle

A significant difference regarding the BMA cell count could be found: The Marrow Cellution needle showed the highest mean cell count compared to the Arthrex and Argon needles (see Tables 2, 3 and 4). Using the same needles (Argon, Arthex) from the conventional technique (Group 1) in the reorientation technique (Group 2) results in a significantly higher viability and a more than double CD34 + cell count.

**Table 3:** Group 2 (reorientation technique): absolute cell numbers were significantly higher than those in Group 1 (conventional technique), regardless of whether the technique was large or small.

Group	Patient #	Orientation	Volume	Centrifuge	Needle	Syringe	Location	Side	Leu/nl	Viability CD34 (%)	CD34 /µl
2	1	Multiple	Big	Angel	Arthrex	Arthrex (2 × 30 ml)	Anterior	Left	12	91	90
2	2	Multiple	Big	Angel	Arthrex	Arthrex (2 × 30 ml)	Anterior	Left	8	94	48
2	3	Multiple	Big	Angel	Arthrex	Arthrex (2 × 30 ml)	Anterior	Left	10	92	80
2	4	Multiple	Big	Angel	Arthrex	Arthrex (2 × 30 ml)	Anterior	Left	11	84	84
2	5	Multiple	Big	Angel	Arthrex	Arthrex (2 × 30 ml)	Anterior	Left	6	73	106
2	6	Multiple	Big	Angel	Arthrex	Arthrex (2 × 30 ml)	Anterior	Left	14	85	207
2	7	Multiple	Big	Angel	Arthrex	Arthrex (2 × 30 ml)	Anterior	Left	9	81	50
2	8	Multiple	Big	Angel	Arthrex	Arthrex (2 × 30 ml)	Anterior	Left	12	88	80
							Group Arthrex	Mean	10 ± 2	86 ± 6	93 ± 47
2	1	Multiple	Small	Angel	Argon	Luerloc 12 ml	Anterior	Right	8	86	90
2	2	Multiple	Small	Angel	Argon	Luerloc 12 ml	Anterior	Right	16	82	124
2	3	Multiple	Small	Angel	Argon	Luerloc 12 ml	Anterior	Right	9	92	103
2	4	Multiple	Small	Angel	Argon	Luerloc 12 ml	Anterior	Right	12	85	102
							Group Argon	Mean	11 ± 3	86 ± 4	105 ± 12
2	5	Multiple	Small	IMPACT	Marrow cellution	Luerloc 12 ml	anterior	Right	16	68	368
2	6	Multiple	Small	IMPACT	Marrow cellution	Luerloc 12 ml	Anterior	Right	20	83	384
2	7	Multiple	Small	IMPACT	Marrow cellution	Luerloc 12 ml	Anterior	Right	14	93	180
2	8	Multiple	Small	IMPACT	Marrow cellution	Luerloc 12 ml	Anterior	Right	16	89	130
							Group Marrow cellution	Mean	17 ± 2	83 ± 10	266 ± 112
Group 2: Significant differences between all groups, see table 4							Mean values of all patients in group 2		12 ± 4	85 ± 7	140 ± 98

There was a significant difference between the three different needles used. The small volume technique had a tendency to outperform the large volume technique.

**Table 4:** Site: there was no difference between the 4 different anatomic sites: anterior, posterior, left, right. Technique: there was a significantly higher cell yield in the multiple orientation technique (group 2) than in the single orientation technique (group 1). Needle: there was a significant difference between the three different needles used. Syringe: there was no significant difference between the different syringes used. Volume: the small-volume technique had a tendency to outperform the large-volume technique in terms of cell number.

Differences in	Group	Table	p values		
			Leu/nl	Viability CD34 (%)	CD34 /µl
**Site**					
Anterior versus posterior^1^	1	2	0.488	0.151	0.423
Left versus right^1^	1	2	0.423	0.657	0.773
**Technique**					
Group 1 versus group^1,2^	**1 and 2**	**2 and 3**	**<0.001**	**<0.001**	**0.003**
**Needle**					
Marrow cellution vs. Argon vs. Arthrex^2^	**2**	**3**	**0.008**	**0.364**	**0.003**
**Syringe**					
16 × Arthrex (2 × 30 ml) vs. 16 × Luerloc (6 × 10 ml)^1^	1	2	0.423	0.657	0.773
**Volume**					
Big (8 × Arthrex 60 ml) versus small (8 × Luerloc 12 ml)					
T-test	2	3	**0.049**	0.988	0.077
Mann–Whitney-test	2	3	0.064	1	**0.018**

Significant values are indicated in bold.

^1^t-test, ^2^ANOVA, Kruskal Wallis test.

### Q3: volume

Smaller volumes had higher cell numbers than large volumes in both groups: Using the reorientation technique (Group 2), 1 × 12 ml led to a significantly higher leucocyte and CD34 + number than using 2 × 30 ml, see Tables 3 and 4. Using the conventional technique (Group 1) 6 × 10 ml led to a higher CD34 + cell count than using 2 × 30 ml.

### Q4: new technique

A reorientation technique with multiple aspirations of 2 ml significantly enhanced the output compared to the conventional technique with a single suction. The reorientation technique (Group 2) leads to a better viability (85 ± 7 versus 75 ± 9%, p < 0.001) and double cell count of leucocytes (12 ± 4 versus 5 ± 2/nl, p < 0.001) and a more than triple cell count of CD34 + cells (140 ± 98 versus 40 ± 40/µl, p = 0.003) compared to the conventional technique of Group 1 (see Tables 2, 3 and 4). This could be demonstrated regardless of the needle and volume.

## Discussion

We treated patients with osteoarthritis of the knee and injected a bone marrow aspirate into the respective knee joint. We evaluated the harvesting of the BMA in a prospective study with three different needles and various volumes of syringes and two different techniques to compare the conventional (single orientation) technique (Group 1) with the reorientation technique (Group 2). We were able to demonstrate that the reorientation technique with the use of multiple small volumes and the reorientation of the needle leads to a significantly higher cell count (p < 0.001), as well as a higher viability (p < 0.001), in the BMA. Furthermore, we evaluated several different needles and assessed two different harvesting sites: anterior versus posterior iliac crest, which makes a difference in daily practice regarding patient positioning.

Regarding the site of harvesting, our findings are consistent with Pierini, who also did not find a significant difference in cell count between anterior and posterior harvesting; however, he found some deviations in colony forming units of unknown clinical relevance^17^. From the clinical point of view, it is an advantage to harvest from the anterior direction because patients can then be operated on in the supine position and do not have to be turned in the lateral or prone position, since the hip and the knee joint are then more easily accessible for the subsequent injection. Furthermore, obese patients may not tolerate the prone position.

The use of a needle with a smaller diameter leads to comparable cell counts but is less invasive for the patient (Argon® versus Arthrex®, Groups 1 and 2). Compared to the other two needles in our study, both of which have suction over the tip, the Marrow Cellution needle with lateral suction showed the highest values of leucocytes and CD34 + cells. The manufacturer claims that the bone marrow aspirate from a Marrow Cellution needle can be used without centrifugation in cases of osteoarthritis: Varady showed significant clinical improvement using a fenestrated trocar and no centrifugation for the treatment of knee osteoarthritis; however, he did not use a control group, and he did not count cells^28^. In contrast, Feddahi compared the Marrow Cellution needle (lateral suction) with the Jamshidi needle (suction over the tip) in 12 patients, both of comparable diameter, and found no difference in cell count and colony forming units^9^. Two issues with the Marrow Cellution needle might hinder regular clinical use: first, the higher invasiveness (longer and larger diameter) and second, the high price compared to the other two needles. Therefore, further clinical studies are necessary to evaluate a possible better clinical effect of a needle with lateral suction at a significantly higher price.

This study is the first to prove that a reorientation technique with multiple small volumes has the following advantages over the conventional technique with a single-orientation suction technique with one large volume: the leukocyte cell count doubles (p < 0.001), and the CD34 + count per μl can be tripled (p = 0.003). Using different needles and different syringes alone does not lead to significant differences in the conventional technique, which is obviously the result of dilution of the syringe with peripheral blood, as described previously^21,29,30^. We can further support these findings with our data as follows: The standard deviation of the mean CD34 + cell counts/µl of the reorientation technique (Group 2) is relatively lower (140 ± 98) than that of the conventional technique (Group 1) (40 ± 40), indicating that the harvesting is more homogenous and reliable and has less interpatient variability. This can be an indicator that in the conventional technique, there is a higher dilution with blood. Oliver also conducted a study of comparable design, but obviously, the number of aspirations in her study was too few to demonstrate a significant difference in cell count^13^. Hernigou also favours the small-volume technique to minimize dilution with peripheral blood^23^. To further assess the effect of large versus small volumes, we clustered patients in Group 2 into 2 subgroups, one in which 60 ml was harvested from the left side (large volume) and the other in which 12 ml was harvested from the right side (small volume); there was a tendency towards a higher cell yield in the small volume technique (t test and Mann–Whitney test).

The newly described reorientation technique of Group 2 has the following advantages in our institution: (1) the mean cell count per patient is higher; (2) the procedure is less invasive (two puncture sites instead of four; no turning from the supine to the prone position); (3) the procedure is approximately three times faster (shorter centrifugation time); (4) anaesthesia is easier in the supine than in the prone position; and (5) no repositioning or turning of the patient is necessary to reach the hip or the knee joint.

The overproportional distribution of men (64%) in our study can be explained as follows: We are a private institution, and stem cell therapy is not reimbursed by insurance companies in our country. Due to the gender pay gap, it is likely that more men are able to choose costly innovative therapies.

It was not the aim of the present study to measure MSCs using cultivation and expansion and to count colony forming units on a regular basis. We assumed that cell counts from BMA were better comparable than CFU counts, since the latter can be biased by many factors from the laboratory. However, a direct link to clinical outcomes has not yet been described, either for MSCs or for CD34 + cells, and this is part of our ongoing studies.

In our study, we used an Arthrex Angel centrifuge, but there are other comparable devices on the market with similar functionality^10,11,31^.

It can be concluded that the reorientation technique outperforms the conventional technique regarding the viability and cell count of leucocytes and CD34 + cells. The use of a cannula with lateral suction instead of suction over the tip can significantly enhance the cell count, albeit at a higher price. For the use of bone marrow aspirate in the case of arthritis, it might therefore be a future option to harvest a maximum cell yield in the reorientation technique and to omit centrifugation.

## Methods

All methods were carried out in accordance with relevant guidelines and regulations. All experimental protocols were approved by the Ethics Committee of the Medical University of Graz (Auenbruggerplatz 2, First Floor, 8036 Graz). Informed consent was obtained from all subjects and/or their legal guardian(s).

In the SUSTEXAP (SUstainable STemcell EXtraction and APplication) study (IRB approval 31–152 ex 18/19), we assessed various methods to aspirate bone marrow and to concentrate the bone marrow aspirate with an Arthrex Angel centrifuge.

Forty-eight bone marrow aspirations were retrieved from patients with grade IV degenerative knee arthritis and compared (see Table 1).

The inclusion criteria were as follows: (1) knee-joint arthritis of grade III/IV (indication for stem cell therapy) and no artificial joint favoured by the patient; (2) age: 18–90 years; (3) no chronic inflammatory disease (rheumatoid arthritis, inflammatory bowel disease, disseminated encephalitis, etc.); (4) no immunosuppressive medication (corticoid, biologicals, specific antibody-therapy, etc.); (5) BMI > 15, BMI < 30; (6) no malignant disease; and (7) no previous operation or puncture of the iliac crest.

We aspirated bone marrow from the iliac crest, and after centrifugation, we injected the bone marrow aspirate into the respective knee joint to improve knee function in the same session without significant manipulation of cells.

### Q1: site

Each patient received at least 2 punctures of the iliac crest under sedoanalgesia with two different sets at the same time to serve as his own control (right versus left, anterior versus posterior, different needles, volumes, techniques); see Tables 1, 2, 3 and 4. All operations were performed by one surgeon (C.P.) in one institution with the same team.

In Group 1, the conventional technique was used to identify any possible differences between the anterior and posterior iliac crest. Therefore, the same technique and needle and syringe were used, anteriorly and posteriorly, respectively. This had the disadvantage of turning the patient from the supine to the lateral position to reach the posterior approach. On the left and right sides, two different systems were compared, so the patient served as his own control. See Tables 1 and 2.

### Q2: needle: three different types of needles were compared

*Argon* needle, 1.5 mm diameter, suction from the tip of the needle. Cost 1 €.

*Arthrex* needle from the original Arthex Angel set, 3 mm diameter, suction from the tip of the needle. This needle is free, since it is already part of the set.

*Marrow Cellution needle*, 3 mm diameter, lateral suction with specific extra holes. Cost 600 €.

### Q3: Different volumes of syringes were compared

“*Arthrex*”: Two syringes of the Arthrex Angel Set with 30 ml each were filled consecutively using continuous aspiration from two adjacent sites with a 10 mm distance, as described in the Arthrex user manual. Harvest output: 60 ml of bone marrow aspirate.

“*Luerloc*”: Six 10 ml Luerloc syringes were filled from 6 punctures, 10 mm adjacent to each other. Each syringe was filled with one slow pull. Harvest output: 60 ml of bone marrow aspirate. With this technique, we aimed to find a difference between a larger volume of the syringe (Arthrex) and a smaller volume (Luerloc), since the number of millilitres after which the dilution with peripheral blood occurs is not yet published. The patient served as his or her own control (left versus right). Harvest output: 60 ml of bone marrow aspirate.

“*Luerloc 12 ml”*: small volume technique: A 12 ml Luerloc syringe was filled from 3 puncture sites, 10 mm adjacent to each other. Each syringe was filled in 2 ml steps with maximum suction force, and after 2 ml, the direction was changed according to the protocol of Christine Olivier. After 4 ml, the next puncture site was taken^13^. Harvest output: 12 ml of bone marrow aspirate.

### Q4: Reorientation techniques versus the conventional technique

Group 1: “conventional technique”: The whole syringe is filled at once (continuous moderate suction force) without reorientation. This is the methodology that most publications describe. We started with Group 1 in this technique to determine whether ventral or dorsal harvesting would make a difference. We compared anterior and posterior harvesting of the same patient, who served as his or her own control. Furthermore, we compared two different syringes and needles in the same patient (left versus right), and in this case, the patient served as his or her own control. See Tables 2 and 4.

Group 2: “reorientation technique: The syringe is filled with maximum suction force and a volume of 2 ml; then, the needle is reoriented in the bone, and another 2 ml is added. After two aspirations (i.e., 4 ml), a new harvesting site, 10 mm adjacent to the iliac crest, was taken, which was repeated three times until the maximum volume of 12 ml was reached. The anatomy and histology in the bone marrow made us assume that this technique would minimize the effect of dilution with peripheral venous blood and thus lead to a higher cell yield. We used the new reorientation technique on both sides and compared different syringes and needles on the left side and the right side of the same patient, who served as his or her own control. See Tables 3 and 4.

Finally, we compared Group 2 with Group 1 (see Table 4).

### Analysis and of the bone marrow aspirate (BMA)

One millilitre of the bone marrow aspirate from each site was evaluated by FACS analysis. Since no specific marker exists to identify mesenchymal stem cells directly without involving isolation and cell culture, we focused on CD34 + cells since these cells contribute to a large proportion of pluripotent stem cells and can be easily detected. Furthermore, we did not focus on CFU assays, since they are not the most quantitative measure of the presence of bone marrow MSCs and are not proven to correlate with clinical outcome^11^. The Arthrex Angel centrifuge was used because it showed the highest concentration increase of CD34 + cells in a previous study compared to other commercially available sets^10^.

**Figure 1:**
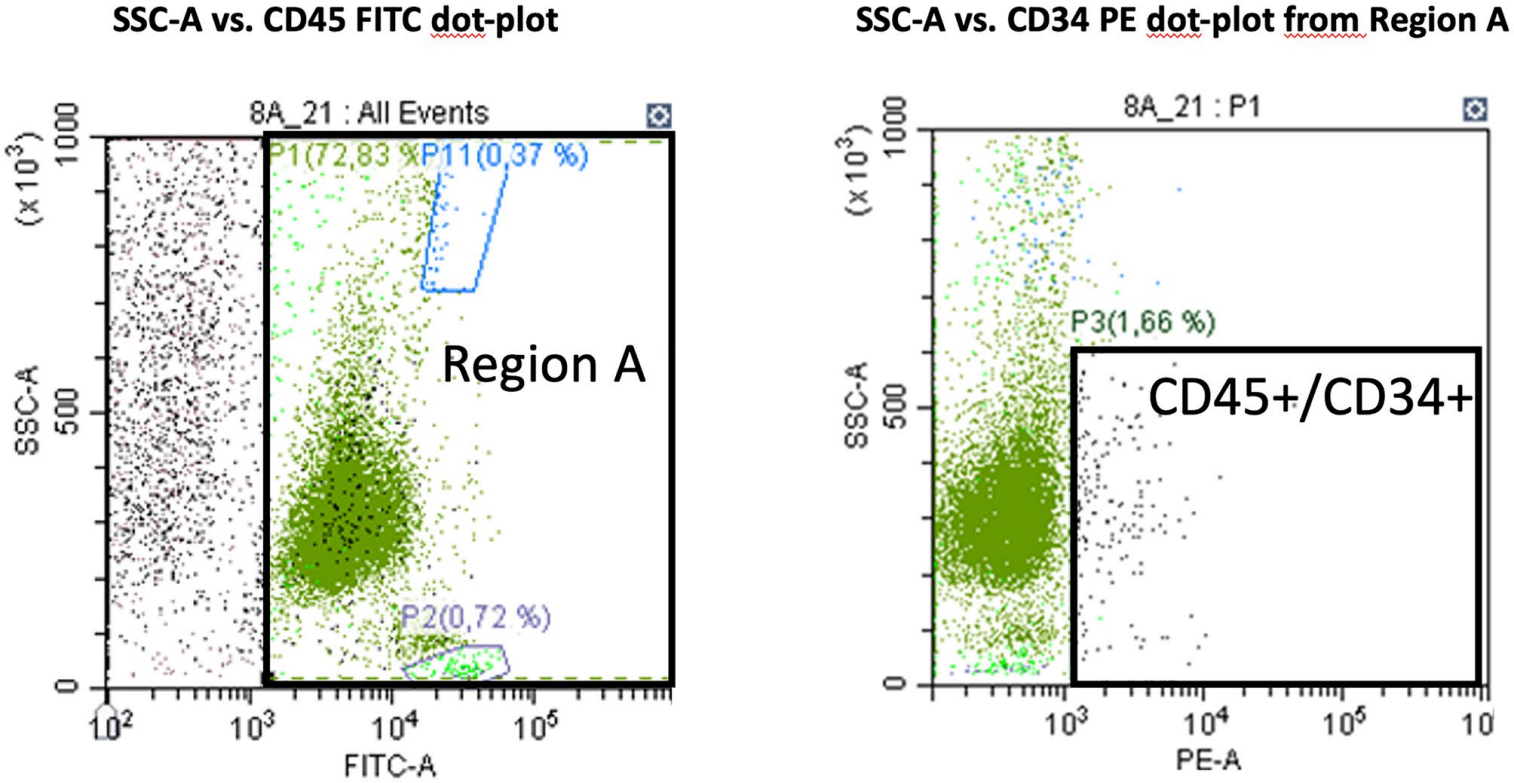
Representative example of the FACS analyses of CD34 + /CD45 + cells.

Analysis was performed with a CytoFLEX Flow Cytometer (Beckman Coulter). Briefly, for each patient’s sample, five tubes were prepared and processed in parallel: (1) single staining with CD34 PE was added to the wash tube; (2) double staining with CD34 PE and CD45 FITC- wash tube; (3) double staining with CD34 PE and CD45 FITC—Trucount tube; (4) isotype control reagent-IgG1 PE and CD45 FITC—wash tube and (5) isotype control reagent-IgG1 PE and CD45 FITC—Trucount tube. To avoid nonspecific staining, the cells were subsequently incubated with 10% (vol/vol) heat-inactivated human AB serum at 4 °C. Tubes were stained and prepared according to the manufacturer’s recommendation. All samples were then stored at 4 °C in the dark and analysed by flow cytometry within 1 h. Laboratory processing techniques followed Good Manufacturing Practice^32^. See Fig. 1.

The total number of CD34 + cells from samples was measured by flow cytometry with Trucount BD. The number of CD34 + cells per microlitre was calculated according to the following formula:

$${\text{CD34+ cell}}/\upmu{\text{l}} = ({\text{no. of CD34+ cells}} \times {\text{bead count per test}} \times {\text{dilution factor)}}/{\text{no. of beads collected}}.$$

The primary outcome parameters were leucocytes (# per nanolitre), viability of CD 34 cells (%), and CD 34 cells (# per microlitre).

The cells collected by the procedure were subsequently centrifuged, and the obtained bone marrow aspirate concentrate was injected into the respective degenerative joint in the same session to minimize time and maximize the viability of cells, following the respective EU-regulations. No significant manipulation was performed (i.e., no addition of growth factors, hormones or cytokines).

Group 1: The conventional technique without reorientation and an Arthex Angel centrifuge were used in all cases (https://www.arthrex.com/de/orthobiologie/arthrex-angel-system). We used a haematocrit of 7% for this study in all patients, as recommended by the manufacturer.

Group 2: Reorientation technique:

Left side: We used the same syringe and needle as those on the left side in Group 1, but with the new technique.

Right side: Two small-volume techniques were compared: In 50% of the cases, a 12 ml Luerloc syringe was used, and in 50% of the cases, a Marrow Cellution needle with lateral suction was used. In both cases, 12 ml of BMA was processed with the IMPACT centrifuge. (https://www.haemo-pharma.at/wp-content/uploads/2019/10/Technisches-Datenblatt-IMPACT.pdf).

### Statistics

To compare the different sites and the two groups, t tests and one-way analyses of variance with adjusted post hoc pairwise comparisons by the Scheffe procedure were performed using IBM SPSS Statistics 26 with a significance level of 5 percent. Due to the rather small sample size in some cases, additional Mann–Whitney U tests and Kruskal–Wallis tests were performed to confirm the results of the t tests and ANOVAs. Since more than one measure was available from each patient, paired analyses were considered. However, because not every measure was available from every patient, the data had to be treated as unpaired. Intraclass correlations in a one-way random model within the up to four measures per patient of Group 1 were between 0.6 and 0.7 for the primary outcome parameters, and bivariate correlations between the two measures per patient of Group 2 were between 0.0 and 0.9 (but could not be calculated overall due to different procedures as described above). Because these intraclass and bivariate correlations are generally not higher or are even somewhat lower than the average intercorrelations between the measures, unpaired analyses seem to be appropriate.
